# Fast Filtration of Bacterial or Mammalian Suspension Cell Cultures for Optimal Metabolomics Results

**DOI:** 10.1371/journal.pone.0159389

**Published:** 2016-07-20

**Authors:** Natalie Bordag, Vijay Janakiraman, Jonny Nachtigall, Sandra González Maldonado, Bianca Bethan, Jean-Philippe Laine, Elie Fux

**Affiliations:** 1 Metanomics GmbH, Berlin, Germany; 2 Biogen Idec Inc., Raleigh-Durham, North Carolina, United States of America; 3 Metanomics Health GmbH, Berlin, Germany; Korea University, REPUBLIC OF KOREA

## Abstract

The metabolome offers real time detection of the adaptive, multi-parametric response of the organisms to environmental changes, pathophysiological stimuli or genetic modifications and thus rationalizes the optimization of cell cultures in bioprocessing. In bioprocessing the measurement of physiological intracellular metabolite levels is imperative for successful applications. However, a sampling method applicable to all cell types with little to no validation effort which simultaneously offers high recovery rates, high metabolite coverage and sufficient removal of extracellular contaminations is still missing. Here, quenching, centrifugation and fast filtration were compared and fast filtration in combination with a stabilizing washing solution was identified as the most promising sampling method. Different influencing factors such as filter type, vacuum pressure, washing solutions were comprehensively tested. The improved fast filtration method (MxP^®^ FastQuench) followed by routine lipid/polar extraction delivers a broad metabolite coverage and recovery reflecting well physiological intracellular metabolite levels for different cell types, such as bacteria (*Escherichia coli*) as well as mammalian cells chinese hamster ovary (CHO) and mouse myeloma cells (NS0).The proposed MxP^®^ FastQuench allows sampling, i.e. separation of cells from medium with washing and quenching, in less than 30 seconds and is robustly designed to be applicable to all cell types. The washing solution contains the carbon source respectively the ^13^C-labeled carbon source to avoid nutritional stress during sampling. This method is also compatible with automation which would further reduce sampling times and the variability of metabolite profiling data.

## Introduction

The metabolome reflects the current biological state of an organism and is the endpoint of all interactions between environment, genome, transcriptome and proteome. The metabolome and its response to different conditions deliver valuable mechanistic insights into as diverse topics as nutrition, diseases, biomarkers, toxicity, crop traits, aging, and stress among others [1–7]. In bioprocessing metabolomics allows pinpointing crucial optimization potential in feeding strategies, nutrient supply, process control and identification of gene targets for improved production quality or capacity [2,8,9].

The current major bottleneck for wide spread use of metabolomics and delivery of highly reproducible and reliable results is the sampling of suspension cultured cells while maintaining the levels of intracellular physiological metabolites unperturbed. Many protocols have been suggested and recently tremendous progress towards sophisticated sampling has been made for various types of organisms such as bacteria [10–16], yeasts [17–20], fungi [21] or animal cells [22–24]. However, each method has different technical limitations or was tested for only one type of cells, e.g. either bacteria or yeast or animal cells. In many cases a comprehensive investigation of the sampling factors affecting the metabolome is missing and a standardized protocol applicable to all types of cells is highly expected [2,24,25].

An optimal sampling should simultaneously (i) remove extracellular metabolite contaminations from culture medium, (ii) avoid loss of intracellular metabolites through leakage, export, unspecific adsorption, interconversions or degradation, (iii) be widely applicable to all types of cells with little adaptation effort, and (iv) ensure optimal subsequent extraction with high recovery rates and broad metabolite coverage [2]. In the case of very rapid sampling (in the range of a few seconds) a compromise between sampling speed or contamination removal has to be found [13].

Sampling strategies consist in either “quench first” (cold/solvent) or “separate first” (filtration/centrifugation). The obvious advantage of “quench first” is the earliest possible stop of metabolism avoiding degradation and interconversion, but literature has shown that not all of the other prerequisites can be achieved for an optimal method. In many cell types quenching leads to considerable leakage, which was reported for the most common quenching method with either solvents (increase membrane fluidity, membrane thinning) [13,21], low temperatures (cold shock phenomenon) [11,16,25] or a combination of both [12,13,15,16,20]. Although “quench first” methods can deliver leakage-free high quality results in specific organisms [19,22,24], they must be thoroughly validated and adapted to each new organism and measurement technique [19,20,24]. The leakage issue can be circumvented when extracellular contaminations are not removed, but then only metabolites with very low to non-existent levels in culture medium can be reliably quantified in cells [26]. However, many metabolites considered as typically intracellular can have substantial extracellular levels, especially when some cell death occurs [12,16,25] and as being part of the applied medium as shown in this study with NS0 cells.

The obvious advantage of “separate first” is the leakage-free removal of extracellular contaminations and the importance of the contamination removal cannot be overemphasized (e.g. in [14,16,18,25] and here presented results). The comparatively long centrifugation times in the range of several minutes renders these approaches unacceptable for important metabolites with fast turnover rates such as nucleotides triphosphates, amino acids or glycolysis intermediates [16,18,22,24–27]. Filtration offers the best compromise between sufficiently fast sampling to reduce degradation or interconversions and leakage-free removal of contaminations as well as being widely applicable to various cell types from bacteria [1,10–13], yeasts [17–19] or fungi [21] through mammalian cells [23]. However, each step during sampling has proved to have its own pitfalls and must be designed with care to achieve robustness. Thus, the following parameters must be critically evaluated (i) filter pore size and type avoiding loss of cells [22] while allowing a quick flow through of the medium, (ii) filter material preventing unspecific adsorption of metabolites leading to low recovery rates and containing low amounts of contaminants impairing subsequent analysis by e.g. high blank levels, (iii) pressure or vacuum strength ensuring fast and leakage-free filtration [12,23], (iv) the nature of the washing solutions and temperature avoiding leakage [12,28] and reducing metabolite interconversions or degradations, and (v) extraction protocols enabling high recovery and coverage [14,26] while simultaneously minimizing metabolite degradation [15]. Unfortunately many suggested protocols provide little details on some of the steps, most often vacuum strength [1,11,12,14,18,20,27] or filter size/material [14,17,18,20,27]. In this study all important steps have been investigated and optimized towards contamination-free, but fast sampling which is easily applicable to all cell types and has been successfully tested with the most common and diverse cell types, bacteria (*Escherichia coli*) and mammalian cells (CHO, NS0).

## Material and Methods

### Ethical statement

**Fig 1:**
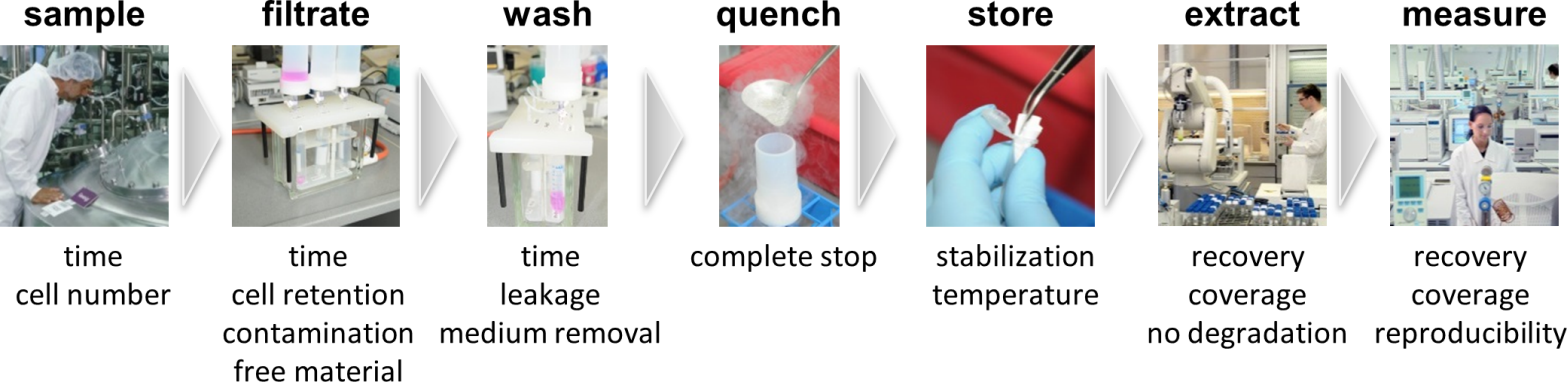
Overview of the steps from sample to peak naming the most critical factors for each step

The individual seen on Fig 1 in this manuscript has given written informed consent (as outlined in PLOS consent form) to publish these case details.

### Cell culture *E*. *coli*

*Escherichia coli* K12 cells Deutsche Sammlung von Mikroorganismen und Zellkulturen (German Collection of Microorganisms and Cell Cultures GmbH) (DSMZ) were cultivated in shaker flasks on 4.5 g/L glucose in LB medium (Sigma Aldrich) under aerobic conditions at 37°C and 220 rpm on an orbital shaker (B. Braun, Certomat BS-1) until late exponential phase.

### Cell culture CHO

CHO-DG44 cells expressing an antibody were cultivated in 160 mL proprietary PBG-CD-C4 media supplemented with 6 mM L-glutamine and start feed in 500 mL Erlenmeyer flasks in an Infors HT multitron cell incubation shaker under 8% CO_2_ at 36.5°C with constant stirring at 150 rpm. Cells were harvested on day 5, cell numbers were 9.0∙10^6^ cells/mL with 93.1% viability as was determined with a Vi-Cell analyzer (Beckman Coulter Inc.) based on trypan-blue exclusion. Medium osmolality on day 5 was 269 mosmol/kg and was measured with a freezing point osmometer (Osmomat 030, Gonotec).

### Cell culture NS0

NS0 cells expressing an IgG antibody were cultivated in a 5 L bioreactor (Applikon Biotechnology, CA) in a proprietary chemically defined medium, consisting of all proteinogenic amino acids, glucose, myo-inositol, O-phosphoethanolamine, pyruvate, putrescine, pantothenic acid, folic acid, glutathione (GSH), nicotinamide, riboflavin, sulfate salts, phosphate salts and trace elements. The production bioreactor process was a 10 day fed-batch process with daily addition of feed starting on day 2. Cells were harvested daily from day 3 on, cell density and viability were determined with a Cedex automated cell counter (Roche Diagnostics Corp., IN). The extracellular content of glucose, glutamate, glutamine, lactate and ammonia were monitored daily with a Bioprofile Flex (Nova Biomedical, MA).

### Cell sampling by centrifugation (C)

Cell suspension was transferred into standard vials and cells were pelleted by centrifugation for 1 min with a standard table top centrifuge (20°C, 14.000 rpm). Supernatant was carefully removed, samples were snap frozen in liquid nitrogen, stored at -80°C and shipped on dry ice.

### Cell sampling by MxP^®^ FastQuench (FQ)

Cell culture suspensions were pipetted onto ethanol pre-wetted standard PTFE filter (Millipore, Fluoropore membrane filter, PTFE, 0.22 μm, 47 mm) placed on a filter funnel (Restek, Discover-47 disk holder) fitted on a filtering manifold (Restek, Resprep). Suspensions were immediately filtered with 35 mbar, applied by a standard vacuum pump (Büchi V-710). Cells were washed with 1 mL of 4.5 g/L uniformly labeled [U-^13^C_6_] D-glucose in isotonic NaCl solution (room temperature) for MxP^®^ Broad Profiling samples and with 4.5 g/L ^12^C-glucose in isotonic NaCl solution (room temperature) for MxP^®^ Energy samples. Once the washing solution flowed through the filtered, liquid nitrogen was immediately added to completely stop metabolism, which was typically around 20 s after withdrawal of cell culture suspensions. Filters were folded and inserted into standard polypropylene vials with forceps and precooled (dry ice) quenching solution was added immediately. Quenching solutions for MxP^®^ Broad Profiling was dichloromethane (DCM) /Ethanol (EtOH) (9:11) and for MxP^®^ Energy DCM/EtOH (2:1). Tubes were frozen in liquid nitrogen and stored at -80°C or shipped on dry ice.

### Metabolic profiling (metabolomics)–MxP^®^ Broad Profiling

The methods are highly standardized, routinely used by metanomics GmbH since 2003 and methodical details were described elsewhere and publications [3,29–32]. In short, gas chromatography (GC) (6890 Agilent) was coupled to a 5973 mass spectrometry (MS) system (Agilent) and liquid chromatography (LC) (1100 Agilent) was coupled to an API4000 tandem MS (MS/MS) System (Applied Biosystems). 5 M ammonium acetate with internal standards in water were added to the cells on the filter in quenching solution and the mixture was homogenized for 5 min in a ball mill (Retsch, Germany). Extracts were spin filtrated (Ultrafree^®^-MC 5.0 μm, Milipore) in 5 min. Filtrates were diluted with DCM, agitated for 5 min with 1400 rpm and phase separation was achieved by centrifugation for 5 min with 12000 rpm. Subsequently, polar and non-polar fractions were separated. For LC-MS/MS analyses, both fractions were dried under reduced pressure and reconstituted in appropriate elution solvents. HPLC was performed by gradient elution using methanol/water/formic acid on reversed-phase separation columns. Mass spectrometric detection technology was applied as described in the patent US7196323 [32], which allows multiple reaction monitoring (MRM) in parallel to a full scan analysis. For GC-MS analyses, the non-polar fraction was treated with methanol under acidic conditions to yield the fatty acid methyl esters derived from both free fatty acids and hydrolyzed complex lipids. The polar and non-polar fractions were further derivatized with O-methyl-hydroxyamine hydrochloride (20 mg/mL in pyridine) to convert oxo-groups to O-methyloximes and subsequently treated with a silylating agent (N-Methyl-N-(trimethylsilyl) trifluoroacetamide) before GC-MS analysis. Data were corrected to internal standards and normalized to the median of reference samples which were derived from a pool generated from aliquots of all sample extracts (once for polar, once for lipid) to account for inter- and intra-instrumental variation.

### Metabolic profiling (metabolomics)–MxP^®^ Energy

UPLC-MS/MS, an Acquity (Waters) coupled to the API5500 a negative mode ESI-MS/MS-System (Applied Biosystems), were used as previously described [30,33]. Samples were kept at all times at 5°C or below. For extraction 1.5 M ammonium acetate and ^13^C-labeled yeast extract as internal standard were added to the cells in the DCM/EtOH quenching solution and the mixture was homogenized for 30 s in a bead mill FastPrep24 (MP Biomedicals) at -20°C. Phase separation was achieved by 2 min centrifugation with 14000 rpm at 4°C. Polar phases were spin filtrated (Ultrafree^®^-MC 5.0 μm, Milipore) in 5 min. Samples were extracted and spin filtrated a second time with additional 1.5 M ammonium acetate. Spin filters were finally washed with water and all three filtrates were combined and lyophilized to remove the ammonium acetate. Samples were resuspended in deionized water and the separation was performed by gradient elution at 45°C using 10 mM tributylammonium acetate (pH 6.2) in A water and B 50% ACN with a gradient from 5–90% B on a 1.5 μm RP UPLC column (VisionHT-HL, 2.1mm×10 cm, Alltech-Grom). All metabolites were measured in MRM mode and each metabolite was normalized against the corresponding ^13^C-analyte.

For production of ^13^C-labeled yeast extract as internal standard a yeast culture of *Candida utilis* (DSMZ sp. 2361) was grown in shake flasks on 10 g/L [U^13^C_6_] D-glucose in yeast nitrogen base amino acid-free medium (Y1250 Sigma–Aldrich). Cells were cultured under aerobic conditions at 28°C on an orbital shaker at 180 rpm and harvested at an optical density of 4 (600 nm, 1 cm cuvette) by short centrifugation at 4°C. Cells were washed twice with 10 g/L [U^13^C_6_] D-glucose in 0.15 M ammonium acetate by centrifugation. The cell pellet was quenched with DCM/EtOH (2/1) and extracted as described for the metabolite profiling MxP^®^ Energy. The polar phase containing the labeled metabolites was stored at -80°C until further use as internal standard.

For selected metabolites with either high turnover rates or of paramount biological interest (glucose-6-phosphate (G6P), fructose-6-phosphate (F6P), fructose-1,6-diphosphate (F1,6P), glutathione (GSH), adenosine monophosphate (AMP), adenosine diphosphate (ADP), adenosine triphosphate (ATP), nicotinamide adenine dinucleotide (NAD), flavin adenine dinucleotide (FAD), glutamine (Gln), glutamate (Glu), pyruvate) quantitative measurements were established based on the MxP^®^ Energy method. Metabolites were quantified via external calibration standards using the relative peak areas to the spiked ^13^C-labeled yeast extract as internal standard which was added to the samples directly before extraction. Linearity was above 0.99 (Pearson R^2^) for ranges from 50 ng/mL to 5 μg/mL, except for pyruvate and ADP where linearity was given in the range of 1 μg/mL to 5 μg/mL.

### Median normalization

Metabolite profiling data were normalized to the median of all metabolites from one platform (pool-normalized ratios for MxP^®^ Broad Profiling and values normalized against the corresponding ^13^C-analyte for MxP^®^ Energy) within each analytical sample. This method assumes that the majority of metabolite levels are similarly influenced by cell densities, which was sufficiently so in this study. In order to keep the high difference between the metabolite levels measured in the two sampling methods an adjustment parameter (median of all MxP^®^ FastQuench (FQ) samples divided by median of all centrifuged (C) samples) was introduced to all C samples.

### Statistical analysis

Metabolite values were log_10_-transformed for all statistical analyses in order to better approximate a normal distribution. For univariate analysis a mixed-effects analysis of variance (ANOVA) model (statistical software R [34] with package nlme [35]) was used to determine culture duration and sampling method induced changes in metabolite abundance. The ANOVA model was set up with the categorical fixed factors day and method, and reactor as random factor (readouts: ratios, p-values, t-values, Benjamini-Hochberg corrected q-values in S1 Table). The ratio can be interpreted as the estimated fold change of one metabolite when comparing one of the factor levels to a reference level, adjusting for all other factors in the model.

For multivariate analysis data was further centered and scaled to unit variance. This introduces a common scale for all metabolites independent of their absolute variance, so that models cannot be dominated by few high-variance metabolites. Multivariate statistical analysis including PCA (principal component analysis) were performed by using Simca P+ software (version 13, Umetrics AB, Umea, Sweden).

## Results and Discussion

The generic workflow from cell to metabolite value of a “separate first” sampling method is to *1*. *draw the sample*, *2*. *separate*, *3*. *wash*, *4*. *quench*, *5*. *(optional) store*, *6*. *extract* and *7*. *measure* (Fig 1). In this study the term sampling method is used for the steps from *1*. *draw* the sample to *4*. *quench*.

While good solutions exist for the steps *1*. *draw the sample* and *4*. *quench*, the steps *2*. *separate* and *3*. *wash* currently have the highest demand for robust and widely applicable set-up. Depending on the aim of a metabolomics study different extraction protocols can be needed and thus an optimal sampling method should be compatible with a wide range of extraction protocols. However, to simultaneously develop a sampling method and test various extraction protocols increases complexity and efforts exponentially. The other option is to apply a rather harsh extraction protocol (here H_2_O/DCM/EtOH with mechanical cell disruption via ball or bead milling) covering broadly both lipid and polar metabolites. A sampling method, which is compatible with such an extraction has a very high probability of being well compatible with similar or milder conditions as well as focused metabolite investigations. Filter stability with other typical extraction solvents can be taken for granted for filters stable in DCM mixtures, ball or bead milling and liquid nitrogen. General metabolite coverage of extractions with multiple solvents is higher than with single-solvent extractions [26].

Manual sample drawing was used as it is the most readily available and easily implemented method without need for special equipment. If an automated sample drawing equipment exists, its use is strongly encouraged and will likely be compatible with the method proposed here. In order to stop all metabolic activity and to minimize impact of subsequent handling variations, a quick freeze in liquid nitrogen was introduced directly in the filter funnel. This is the fastest, most robust and widely available possibility for stopping metabolism. The addition of the ^13^C-labeled carbon source to the washing solution was found to avoid metabolic changes due to nutritional stress.

### Filters

The filter itself is one of the most crucial parameters in filtration sampling methods and must simultaneously be (i) solvent resistant, (ii) contamination-free during sampling and extraction, (iii) mechanically stable in liquid nitrogen and bead mill extraction, and (iv) able to retain all possible types and sizes of cells. In order to retain even small cell types such as bacteria, pore size as routinely used for sterile filtration, which is around 0.2 μm, is necessary. However, small pore sizes can be more demanding towards the filter with higher risk of clogging and rupturing. Therefore, initially also pore sizes up to 3 μm were tested but discarded later on in the qualification process (see further section filtration). The suitability of several commercially available filters were tested according to the above-mentioned criteria in the order of increasing effort. Filters were excluded from subsequent analysis if they disintegrated during extraction, broke after liquid nitrogen application or too inflexible for folding into tubes, left visible residues after GC derivatization of a blank extraction (which would risk clogging the columns or too high blanks) or had high blank levels in either of the applied measurement platforms. Different filter types such as depth or membrane filters as well as different filter materials such as cellulose mixed ester (cellulose nitrate and–acetate) (CME), polycarbonate (PC), polyvinylidene fluoride (PVDF), polyethersulfone (PES), polytetrafluoroethylene (PTFE), nylon or glass fiber were tested. Hydrophilic filters were activated in distilled water and hydrophobic filters in EtOH to enable quick filtration of cell suspensions (filters and tests are detailed in S2 Table). Cell integrity was unaffected by residual distilled water or EtOH in the filters due to the comparatively low amounts and quick wash out with filtrate during filtration. For all subsequent experiments the Fluoropore^TM^ filters were used, as they met all criteria and are world-wide available.

### Filtration

Filtration is the process of applying a pressure difference across the filter material, which drives the separation of particles (e.g. cells) depositing on the filter as filter cake from the fluid (e.g. cell culture medium) which passes through the filter and becomes the filtrate [36]. For sampling purposes, filtration should ideally have a high filtrate flux to minimize time and leave deposited cells undamaged to avoid leakage of intracellular metabolites.

Alterations of biological parameters such as cell size, cell form, cell density, cell age, pH, salinity, viscosity, temperature, extracellular matrix or protein content, to increase filtrate flux [36] directly contradict the aim of an unperturbed, widely applicable sampling method and were not investigated in detail. Adjustable technical parameters influencing filtrate flux are pore size, filter type (membrane or depth), filter material, pressure difference [36] and ratio of deposited cells to filter area.

However here, the technical parameters pore size, filter type and filter material are fixed by the need to (i) retain all possible cell types (pore size around 0.2 μm) and (ii) compatibility with subsequent extraction and measurements (hydrophobic PTFE membrane filter). Filtrate flux increases with decreasing ratio of cells per filter area, but the increase is confined by the need for sufficient cell mass for measurements and still good to handle filter sizes. In order to achieve some safety margin allowing also filtration of non-ideal cell suspensions relatively large filters with a 47 mm diameter were selected. These filter sizes with the small pore size of 0.2 μm worked very well with the required cell numbers ranging from 1-10∙10^6^ of eukaryotic cells (e.g. CHO, NS0) to 1-10∙10^9^ of prokaryotic cells (e.g. *E*. *coli*) per measured sample.

**Fig 2:**
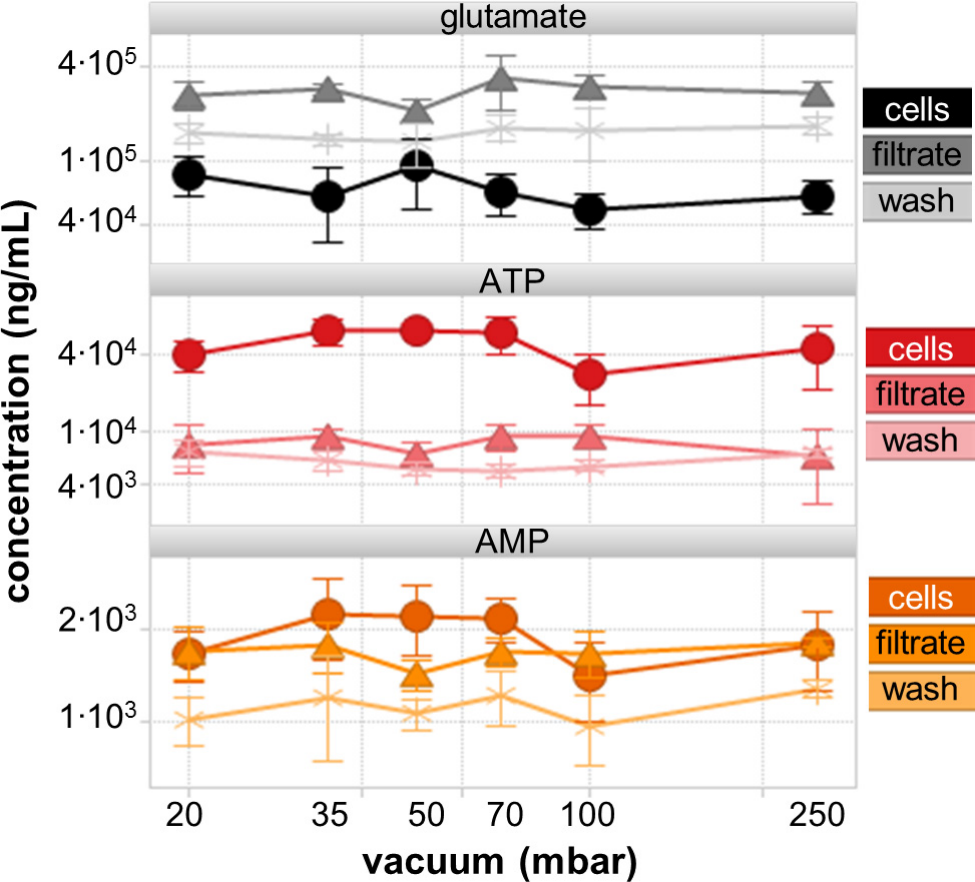
Impact of vacuum strength on glutamate, ATP and AMP levels in CHO cells, in filtrate or in washing solution was low (5 replicates, all scales are logarithmic). All other quantified metabolites (GSH, NAD, ADP, G6P) exhibited similar curves and are not shown for the sake of clarity. The example metabolites were selected due to their biological interest, size difference, cell culture medium content (e.g. glutamate) or in literature described possible leakage [25]. Metabolite level changes compared to 35 mbar were not statistically significant (Student’s t-test, all p values above 0.05).

The increase of pressure difference increases the filtrate flux but at the same time intensifies filter cake compression and thus mechanical strain on cells. Additionally, filter cake compression increases filter cake resistance so that filtrate flux improvement is finite. Impact of vacuum strength ranging from strongest vacuum with 20 mbar to weakest with 250 mbar was tested on cell integrity with CHO cells, which are typical eukaryotic cell and mechanically least stable compared to other cell types such as yeast or bacteria. Within the applied vacuum range metabolite levels in cells and in filtrate remained unchanged suggesting sufficient cell integrity. The slight decrease of some metabolite levels from 35 mbar to 20 mbar (Fig 2) could indicate onset of leakage, but the decreases were not statistically significant (Student’s t-test, all p-values were above 0.05). Most likely the combination of tubing, filtration manifold and comparatively small pore size reduces the mechanical strain finally exerted onto cells, explaining the difference to reported concerns and observations [22]. When the filtration equipment was used in different laboratories, vacuum of ≤30 mbar was found to be difficult to achieve as vacuum efficiency depends also on quality of tubing and seals and. Thus 30 mbar can be seen as the lower limit of routinely usable vacuum strength of the proposed equipment.

Weaker vacuum was investigated to avoid unnecessary strain on cells and values ≥70 mbar resulted in high residual fluid in the filter cakes. The residual fluid formed a rigid ice layer during quenching with liquid nitrogen preventing subsequent folding without material losses. Additionally, sufficient filtrate flux allowing filtration of sufficient cell numbers in less than 5 s was only achieved with vacuum strengths <70 mbar. In conclusion, a vacuum strength of 35 mbar was selected as it was easily reproducible in other laboratories and was the best compromise between avoiding leakage and sufficiently fast as well as dry filtration of diverse cell suspensions.

### Washing

**Fig 3:**
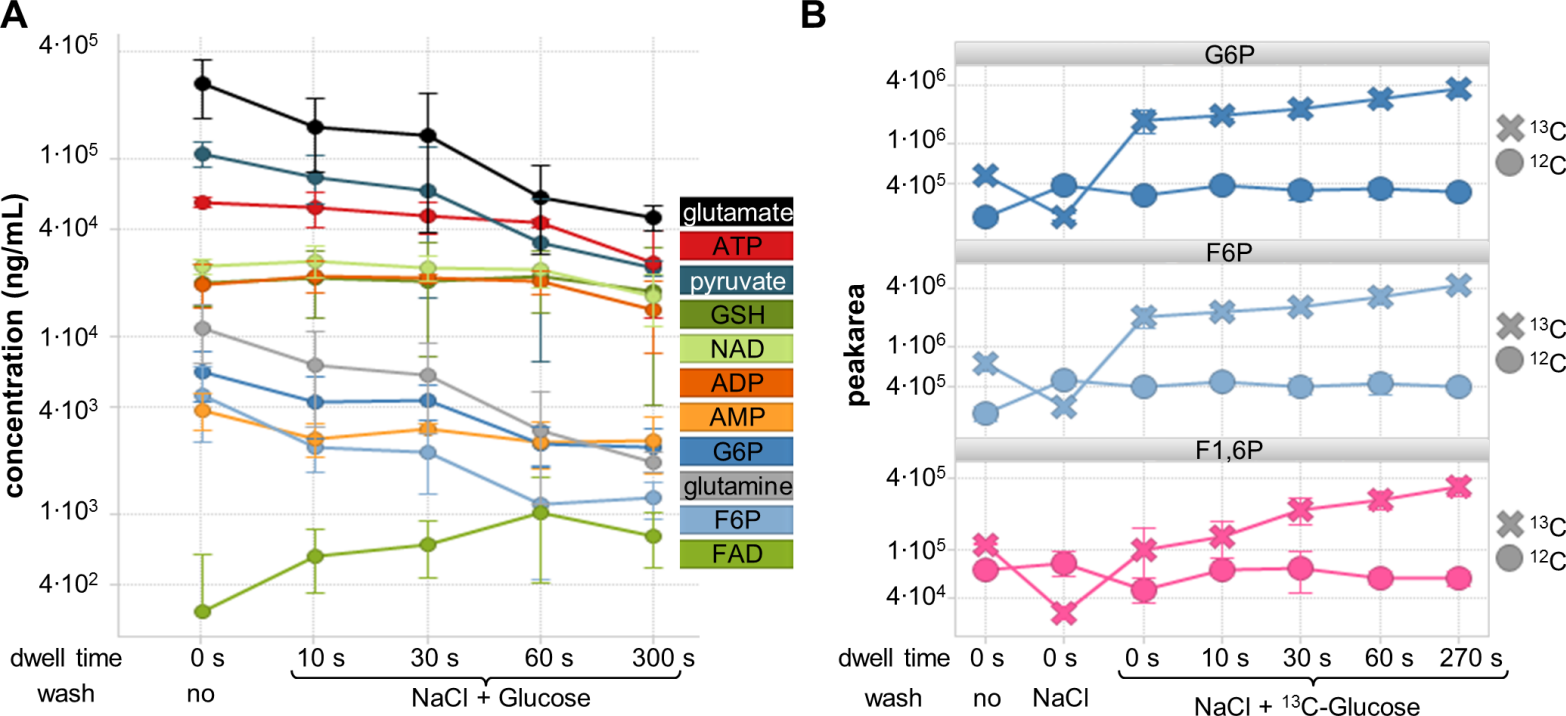
Impact of washing, dwell time and carbon source in washing solution on metabolite levels. A: Impact of dwell time of CHO cells on filter to evaluate impact of delays during sampling (3 replicates). Metabolite levels decreased over time, especially after 30 s for G6P, F6P, glutamine, glutamate and pyruvate, y-axis logarithmic. B: Impact of ^13^C-glucose as washing solution on *E*. *coli* glycolysis metabolite levels. Glucose metabolization of ^13^C-glucose during washing increases ^13^C glycolysis metabolites with very high turnover rates and thereby stabilizes intracellular levels of the corresponding ^12^C metabolites for up to 60 s.

Washing steps aim at the removal of contaminations of the cells from the culture medium. The washing step also increases the overall sampling time and thus the risk for interconversions of metabolites with high turnover rates [16,18,22,24–27]. Therefore, the impact of omitting washing and prolonged sampling time, simulated by letting cells dwell on filters for specific times, was investigated (Fig 3A). Metabolites known to be nutritional supplements and thus with high extracellular levels, e.g. glutamate, glutamine and pyruvate, as well as metabolites that are not commonly expected in the extracellular medium, e.g. G6P, AMP and F6P, exhibited misleading high concentrations when washing was omitted (Fig 3A). One washing step during manual sampling with 1 mL takes about 10 s, so that the lower values observed after washing could also result from degradation or interconversion. However, some of the exclusively intracellular metabolites with known high turnover rates, e.g. ATP, ADP, NAD or GSH showed mostly constant levels for up to 60 s of total sample handling duration in CHO cells. Thus, most of the observed decreases of metabolite level after washing results from the removal of contaminations rather than degradation or interconversion. Additionally, energy charges calculated as ATP+0.5ADP/(ATP+ADP+AMP) [37] were in accordance with published literature [8,37]. However, the energy charge seems to be an insensitive parameter because energy charges were found to be stable within the first 60 s in CHO cells (no wash 0.83; with wash 10 s 0.82; 30 s 0.81; 60 s 0.81; 300 s 0.77; all ±0.02). Other metabolites such as glutamine, glutamate, pyruvate, F6P and G6P showed significant decreases already after 30 s indicating the importance of a controlled and short washing time. Washing with double volume (2 mL) increased handling time and achieved only minor further removal of nutritional supplements (glutamate, glutamine, pyruvate each 4 percentage points of non-washed levels). However, variability increased significantly from median RSD of 20% for 1 mL washing to 55% for 2 mL and levels of metabolites with high turnover rates decreased more pronounced while levels of NAD and FAD increased (energy charge 0.80). Washing volumes should be balanced between sufficient contamination removal and fast performance but it is imperative to measure contamination-free, intracellular metabolite levels. Therefore, washing volume should be selected carefully. Our results suggest that a 1 mL washing volume provides sufficient clean-up for subsequent metabolite profiling analysis.

Composition and temperature of the washing solution is important in order to minimize stress-induced metabolite leakage or release from cells. Cold washing solutions reduce cellular metabolism but also induce the cold shock phenomenon, which is the release or leakage of metabolites due to rapid temperature reduction. The cold shock can occur in any cell type [38] and is well documented for bacterial cells causing leakage of e.g. up to 30% amino acid [25]. Washing of cells with non-isotonic solutions was reported to induce leakage or metabolite release likely due to the osmotic shock, e.g. for gram negative bacteria [12]. Occurrence of leakage or metabolite release seems to occur only after sufficient contact time of cells with the non-isotonic washing solution since very fast (<2 s) washing of adherent mammalian cells with deionized water did not induce any leakage of metabolites from the cells [28]. Washing with water would have the advantage of lowering sample salt content and reducing ion suppression during subsequent MS analysis but carrying out the washing step in a 2 s time frame is unrealistic when manual sampling is required. In contrast, washing with isotonic solutions increases robustness of the sampling method towards some time variations, safely avoiding osmotic shock. In addition, knowledge about time frames for metabolite release or leakage under osmotic shock is limited to a few cell types and other cell types might show faster reactions. Therefore, isotonic washing solutions (NaCl) at room temperature were selected to minimize leakage or metabolite release and thus achieve robust, broad applicability to different cell types. Other salts could also be used if needed, but should be compatible with the analytical instrumentation used for the analysis.

Often the metabolites adjacent to the main carbon source are the ones with the highest turnover rates, such as glycolysis intermediates glucose-6-phosphate (G6P), fructose-6-phosphate (F6P) and fructose-1,6-diphosphate (F1,6P) for glucose. Stabilizing these metabolites during the washing is of high interest, especially for cell types with known fast metabolizations rates such as *E*. *coli*. Washing with ^13^C-glucose leads to metabolization of ^13^C-glucose during washing and consequently increases ^13^C glycolysis metabolites with very high turnover rates. The ^13^C metabolization simultaneously stabilized intracellular levels of the corresponding ^12^C metabolites for up to 60 s (Fig 3B) and did not interfere with ^12^C metabolite measurements. Levels of ^12^C AMP, FAD and NAD were found similar for washing with or without ^13^C glucose with a weak trends towards increased levels after 60 s (not significant, Student’s t-test, all p-value were above 0.05). Therefore, addition of a ^13^C carbon source to the washing solution in equal concentrations as culture medium is recommended to preserve ^12^C-metabolite levels especially when fast turnover rates are expected.

### Metabolic stop, quenching and stabilization

After washing, the quickest and most efficient way to stop metabolic activity is to snap freeze with liquid nitrogen by pouring it directly on the cells immediately after filtering the washing solution. This also eliminates influence of possible time variations from the subsequent folding of the filter into tubes and addition of quenching solution, which are comparatively the most variable and lengthy steps (sampling in teams of two persons is easier). After filters were folded into tubes, dry-ice precooled solvents were added to the cells to protect metabolites from oxidation, avoid further metabolic reactions during storage and stabilize metabolites against possible temperature fluctuations during sample shipment. The addition of solvents prior to shipment was found to be far more practical than with inert gas as it also risks accidental loss of cells due to the gas stream. The solvents were selected to correspond to the extraction solvents so that subsequent extraction could be performed without any additional transferring steps such as evaporation and resuspension.

### Application to new cell type (NS0) without additional method adaptation

The transferability of the method was tested with NS0 cells in a different laboratory with personnel following the protocol described above and without extra training in the method. NS0 cells producing an antibody were selected as an example for a typical production system and were grown in a chemically defined medium for ten days with a daily feed starting on day 2 in 4 replicate reactors. MxP^®^ FastQuench (FQ) was applied successfully, with only some sporadic clogging observed towards the end of culture when product titer increased and total cell numbers were high (>20∙10^6^), which is in agreement with the literature [36]. For comparison, cells were also harvested with the simplest method, centrifugation without washing (C). This is also in terms of metabolite profiling the least suitable method due to prolonged handling time allowing degradation and omission of washing steps leading to contamination with cell culture medium.

A total of 255 metabolites were found at concentration levels above the limit of quantification (MxP^®^ Broad Profiling and MxP^®^ Energy) for both sampling methods from which 180 were known metabolites and 75 known unknown metabolites. The metabolites covered most important biological classes such as amino acids, energy metabolites, carbohydrates, vitamins, cofactors, nucleotides, complex lipids and fatty acids.

**Fig 4:**
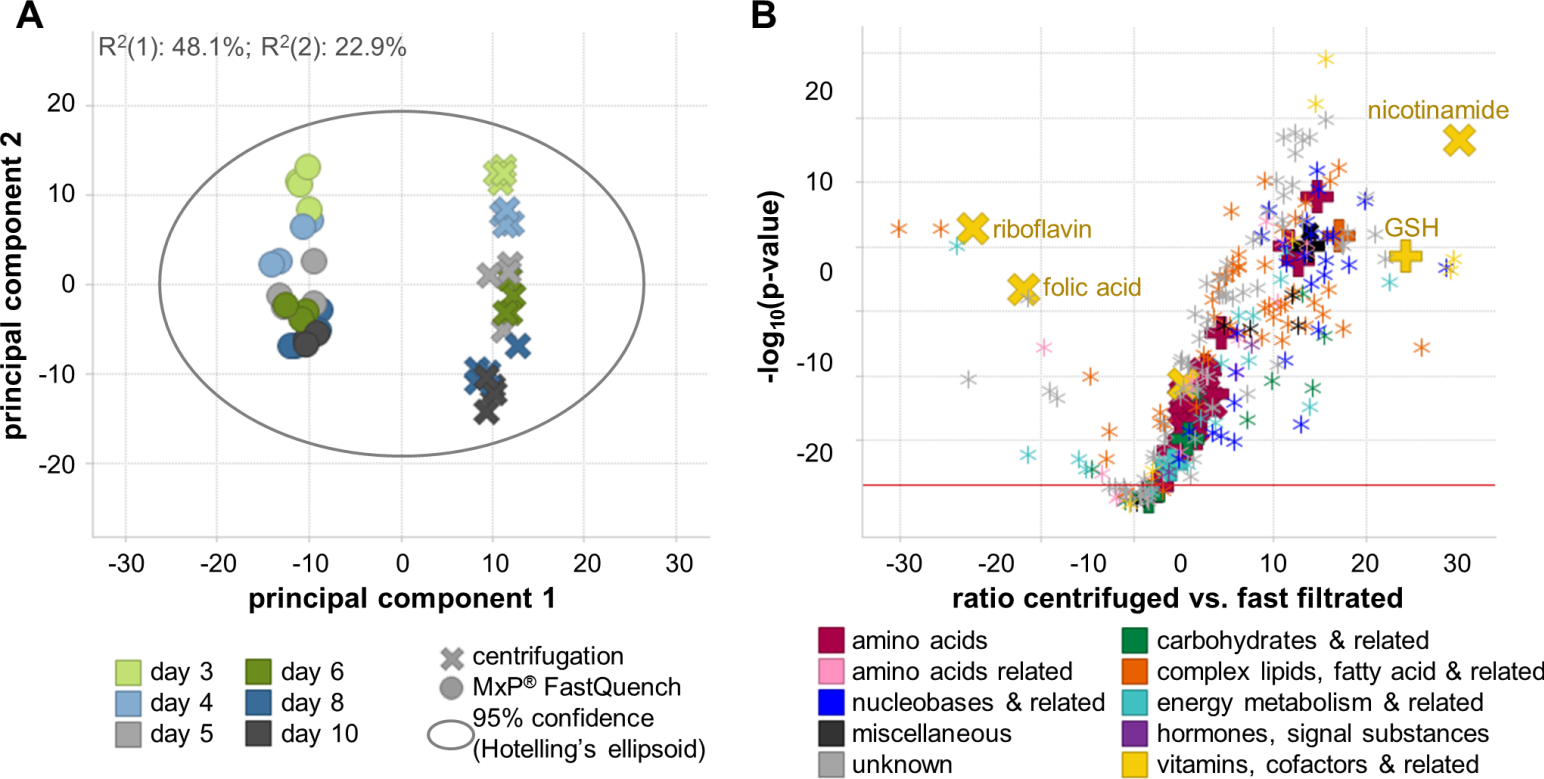
Comparison of metabolite profiles of NS0 cells following sampling with MxP^®^ FastQuench (FQ) versus centrifugation (C) without washing. A: PCA of all samples showing very high difference in metabolite levels between both sampling methods. B: Volcano-plot of ANOVA comparison (all C samples versus all FQ, corrected for culture duration) demonstrating that most metabolites had significantly higher levels in centrifuged samples than in filtered. Pluses mark supplemented metabolites and crosses essential supplemented metabolites.

Multivariate analysis by PCA allows fast identification of trends, patterns and groupings among samples and variables. The PCA model including all samples from both sampling methods clearly demonstrates the extreme differences between C and FQ (Fig 4A). The difference between sampling methods dominates the first principal component which already explains 48.1% of the total variability. Culture duration, which is typically the strongest biological factor, is visible only in the second component, which describes only 22.9% of the remaining variability. The univariate analysis with ANOVA revealed that almost all metabolite levels differed significantly between the two sampling methods (224 of 255 metabolites with a Benjamini-Hochberg corrected q-value<0.05). A total of 206 metabolites exhibited higher levels in C samples than in FQ and only 18 metabolites had lower values (Fig 4B, S1 Table). From these 18 metabolites, 14 s were known metabolites and were suitable for active or preferential cellular uptake and metabolization such as glycolysis intermediates (dihydroxyacetone phosphate, phosphoenolpyruvate), citrate cycle intermediates (α-ketoglutarate, succinyl- coenzyme A), vitamins (folic acid, riboflavin), phosphatidylcholines, monosaccharides (arabinose).

From the metabolites supplemented in the feed, 31 were measured and 28 thereof were found to be significantly higher in C samples, one with similar levels (myo-inositol) and two essential vitamins (folic acid, riboflavin) with significantly lower values than in FQ samples (Fig 4B). Thus, C samples must be severely contaminated with cell culture medium and the two essential vitamins were seemingly under dosed and may be depleted from the cell culture medium.

The fact that most of the measured metabolites were significantly higher in C samples than in FQ samples also shows how complex the supernatant of cells grown in chemically defined medium can become within three days of inoculation. Cell death and active secretion of intracellular metabolites has been discussed previously in literature [16,25] and the extent as well as impact on results can be seen clearly in this work. Degradation or interconversion of metabolites has to be expected during the longer handling times for C samples but is masked by the contamination. The impact of contamination with cell culture medium was much stronger for NS0 cells than in the results presented above for CHO or *E*. *coli* due to the higher cell death rates for NS0 cells.

The metabolites detected with lower levels in C than in FQ samples also suggest that these metabolites may be preferentially used by the cells and are not adequately fortified in the medium, thus making them for supplementation potentially increasing cell growth. Some of these metabolites are already well known to be beneficial when supplemented such as α-ketoglutarate [39] or lipids [40].

**Fig 5:**
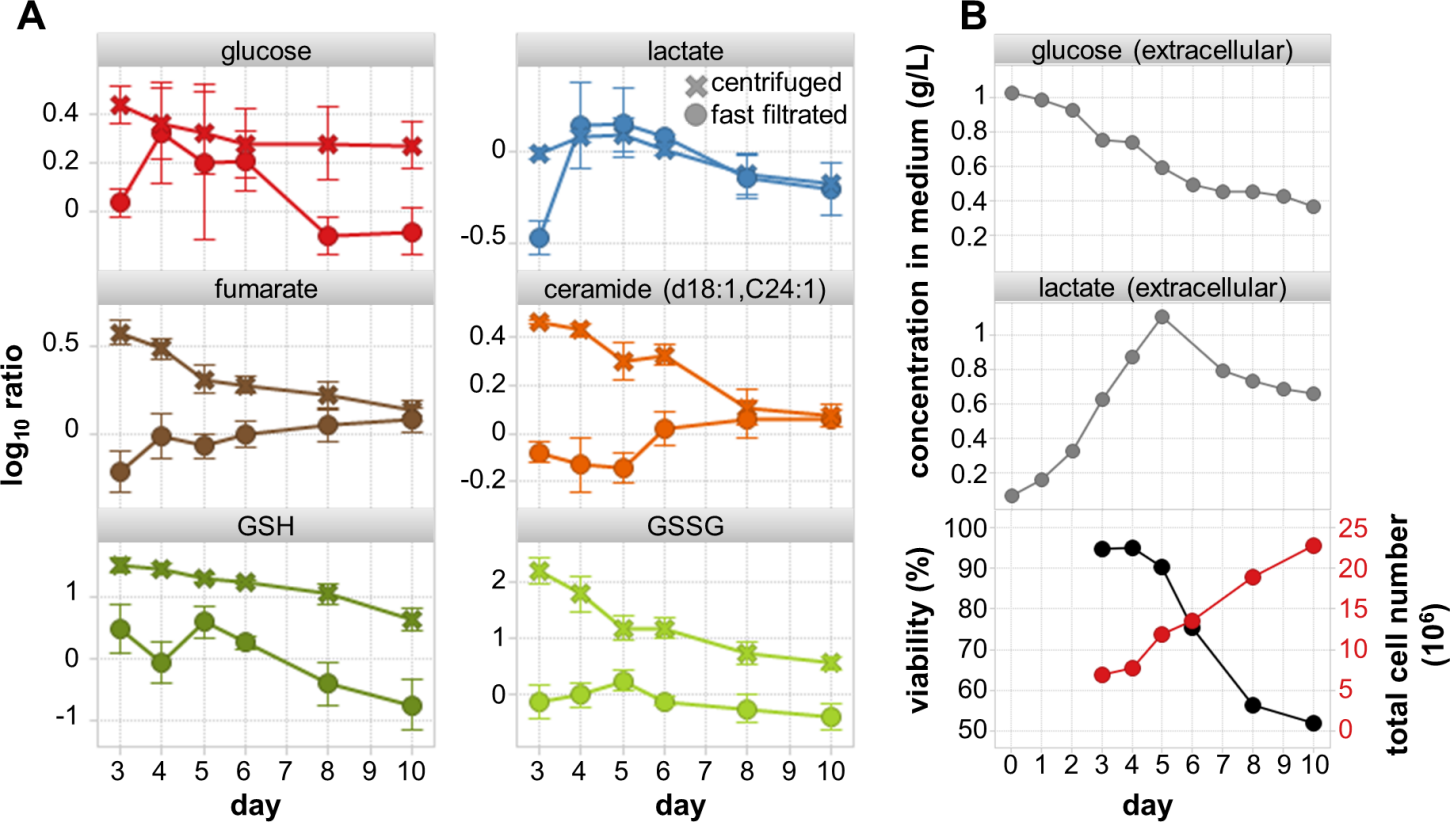
A. Comparison of metabolite levels for centrifuged (no washing) in contrast to MxP® FastQuench (with washing) samples of NS0 cells. Impact of sampling on metabolite levels for selected metabolites with especially misleading results for centrifuged (no washing) in contrast to MxP^®^ FastQuench (with washing) samples. B Extracellular levels of glucose and lactate as well as cell viability and total cell number for comparisons

The contamination with cell culture medium of centrifuged samples results in very misleading interpretations, often not only metabolite levels between sampling methods differed but also the progression of metabolite levels with culture duration differed considerably (Fig 5A).

For example, intracellular glucose and lactate levels in C samples would seem to be rather constant and only decrease slightly in the later days. The lactate to glucose ratio remained constant in the range 0.4 to 0.6. In contrast, FQ samples suggest lower glucose and lactate levels at day 3 (exponential growth phase) indicating efficient glycolysis into lactate paired with efficient secretion. Accordingly the lactate to glucose ratio of 0.3 is very low and increases with culture duration up to 0.9 which correspond better to expectations and observed extracellular levels of glucose and lactate (Fig 5B). FQ samples suggest low fumarate concentrations which increase during the process and are in line with literature [41] whereas C samples suggest decreasing levels. The third example is intracellular redox status characterized by the ratio of the intracellular antioxidant GSH to its oxidized dimer form glutathione disulfide (GSSG) (Fig 5A). The GSH to GSSG ratio for C sample increased from 0.2 to a maximum of 1.7 at day 8 while for FQ samples the ratio started from 3.2 and continuously decreased to 0.5 at day 10 (ratios calculated from pool-normalized data can only be compared within the same data set). A decrease of the GSH to GSSG ratios is connected to cellular toxicity and cell death [42]. Therefore the decrease in FQ samples toward the end of cultivation is well in line with the decreased glucose availability and increased cell death (Fig 5B) suggesting a lower intracellular anti-oxidative capacity. Another example are ceramides which are known to trigger apoptosis by forming lipid rafts for the clustering of "death receptors", hence facilitating caspase-8 activation and increasing mitochondrial outer membrane permeability and cytochrome c release [43]. In C samples ceramide levels unexpectedly decreased while ceramide levels increased in FQ samples with progressing incubation time (Fig 5A) which agrees well with the increasing number of dead cells (Fig 5B).

Thus, contamination with cell culture medium has a high impact and risk misleading results as early as day 3 of cell culture, even when the cell viability is greater than 95%. Special care and caution should be taken in very rapid sampling in the sub-second range where washing has to be omitted to achieve sufficient speed, e.g. by determination of extracellular levels and mass balancing for every investigated metabolite.

## Conclusions and Outlook

In this study the most critical factors for each step during sampling for metabolomics analysis were determined (Fig 1) and accordingly a very robust fast filtration sampling method combined with a washing solution that preserves the metabolic state, MxP^®^ FastQuench was developed.

The sampling method–established and validated for prokaryotic (*E*. *coli*) and eukaryotic (CHO) cells was successfully applied for NS0 cells at a different laboratories with staff who was not familiar with the method. Cell size and form are the main factors determining the retention of cells on filters [36]. The selected filter with a pore size of 0.22 μm was found to be suitable to all cell types.

As test experiments we recommend to determine, e.g. with 5 different cell numbers, the optimal amount of cells that can be well filtered with the selected filtration assembly, applied vacuum (50 mbar to 35 mbar) and cell culture type without risking clogging. For 47 mm filters cell numbers ranging from 1-10∙10^6^ for eukaryotic cells (e.g. CHO, NS0) and 1-10∙10^9^ for prokaryotic cells (e.g. *E*. *coli*) per filter are a good working range and for less cells filter diameter can be accordingly reduced. Second, we recommend performing once the full sampling and measurement of 2 blanks, 5x replicates with isotonic washing and 5x replicates with isotonic washing plus ^13^C carbon source, measuring both cells and final filtrates.

Other extraction protocols can be expected to work well as long as care is taken to avoid unnecessary metabolite degradation from elevated temperatures, prolonged handling or repeated evaporations [15] and allow sufficient metabolite recovery [14,26]. The cells are pressed into a filter cake during filtration and thus attain some additional mechanical robustness so that strong mechanical cell disruption should be applied such as steel bead milling to ensure complete metabolite release. The solvent added for sample quenching during storage or transport should be water-free and compatible with the subsequently planned extraction method to avoid additional evaporation steps.

Automatization of fast filtration would further improve sampling speed and results. Although automation is always preferable, the instrumental demand is much higher and may not be worthwhile especially for sporadic users.

The sampling method presented here is straightforward and relies on readily available equipment allowing its use in different labs. Additionally, the developed fast filtration method, MxP^®^ FastQuench, is compatible with high-throughput, routine extraction and measurement protocols used for sample types such as plasma, blood, or urine, thereby leading to higher comparability of results between these different matrices.

## Supporting Information

S1 Table ANOVA read out comparing all centrifuged samples versus all fast filtrated samples. (XLSX)

S2 Table Key characteristics of the filters and the consecutively tested criteria are summarized. (DOCX)
