# Supplementary material for: Fast Filtration of Bacterial or Mammalian Suspension Cell Cultures for Optimal Metabolomics Results
Source: PLoS One. 2016 Jul 20;11(7):e0159389. doi: 10.1371/journal.pone.0159389 (PMC4954723; doi:10.1371/journal.pone.0159389)
Supplement: S2 Table — (DOCX) [file pone.0159389.s002.docx]

**Fast filtration of bacterial or mammalian suspension cell cultures for optimal metabolomics results**

Natalie Bordag^1^, Vijay Janakiraman^2^, Jonny Nachtigall^1^, Sandra González Maldonado^1^, Bianca Bethan^1^, Jean-Philippe Laine^3^, Elie Fux^1*^

^1^metanomics GmbH, Berlin, Germany; ^2^Biogen Idec Inc., Raleigh-Durham, North Carolina, United States, USA; ^3^Metanomics Health GmbH, Berlin, Germany

*corresponding author: [elie.fux@metanomics.de](mailto:elie.fux@metanomics.de), +49 30 34807 174

S2 Table: Key characteristics of the filters and the consecutively tested criteria are summarized. All filters had a diameter of 47 mm and tests were performed in duplicates. Hydrophobic filters were activated in EtOH, hydrophilic in water.

| **filter** | **pore** | **material** | **polarity** | **type** | **extraction**  **resitant** | **liquid N_2_**  **stable** | **residue-free after**  **GC derivatization** | **low blank** |
| --- | --- | --- | --- | --- | --- | --- | --- | --- |
| glas fiber filters type A/E,  Pall^®^ Life Sciences, VWR | 1 µm | borosilicate glass without binder | hydrophilic | in-depth | no |  |  |  |
| glas fiber filters type A/B,  Pall^®^ Life Sciences, VWR | 1 µm | borosilicate glass without binder | hydrophilic | in-depth | no |  |  |  |
| glas fiber filters type A/C,  Pall^®^ Life Sciences, VWR | 1 µm | borosilicate glass without binder | hydrophilic | in-depth | no |  |  |  |
| glas fiber filters type A/D,  Pall^®^ Life Sciences, VWR | 3 µm | borosilicate glass without binder | hydrophilic | in-depth | no |  |  |  |
| Metrigard^TM^ filters,  Pall^®^ Life Sciences, VWR | 0.5 µm | glass fiber with acrylic binder | hydrophilic | in-depth | no |  |  |  |
| Glass Fibre (GF) Prefilter, Sartorius AG | 0.2 µm | glass fiber with acrylic binder | hydrophilic | in-depth | no |  |  |  |
| LCR filters, Millipore^®^ Corporation | 0.5 µm | PTFE | hydrophilic | membrane | yes | no |  |  |
| Nylon filter, Carl Roth GmbH | 0.45 µm | Nylon | hydrophilic | membrane | yes | yes | no |  |
| Roth PES-Membranfilter | 0.22 µm | PES | hydrophilic | membrane | yes | yes | no |  |
| PES filter, Sartorius AG | 0.45 µm | PES | hydrophilic | membrane | yes | yes | no |  |
| PC track-etched, Sartorius AG | 0.2 µm | PC | hydrophilic | membrane | yes | yes | no |  |
| Durapore**^®^**, Millipore^®^ Corporation | 0.45 µm | PVDF | hydrophobic | membrane | yes | yes | no |  |
| Omnipore^®^, Millipore^®^ Corporation | 0.45 µm | PTFE | hydrophilic | membrane | yes | yes | no |  |
| CME filter, Carl Roth GmbH | 0.45 µm | CME | hydrophilic | membrane | yes | yes | yes | no |
| Immobilon-NC, Millipore^®^ Corporation | 0.45 µm | CME | hydrophilic | membrane | yes | yes | yes | no |
| Durapore**^®^**, Millipore^®^ Corporation | 0.22 µm | PVDF | hydrophilic | membrane | yes | yes | yes | no |
| PTFE, Sartorius AG | 0.2 µm | PTFE | hydrophobic | membrane | yes | yes | yes | no |
| **PTFE type SU, Pieper Filter GmbH** | **0.2 µm** | **PTFE** | **hydrophobic** | **membrane** | **yes** | **yes** | **yes** | **yes** |
| **Fluoropore^TM^, Millipore^®^ Corporation** | **0.22 µm** | **PTFE** | **hydrophobic** | **membrane** | **yes** | **yes** | **yes** | **yes** |

PC – polycarbonate, PTFE - polytetra-fluoroethylene, PES – Polythersulfone, CME – cellulose mixed ester (cellulosenitrate and –acetate)
